# Gold Versus Rubber for Artificial Dentures

**Published:** 1881-05

**Authors:** I. N. Carr

**Affiliations:** Tarboro’, N. C.


ARTICLE II.

### GOLD VERSUS RUBBER FOR ARTIFICIAL DENTURES.

BY DR. I. N. CARR, TARBORO', N. C.

Physicians often find it necessary to prescribe a set of teeth for their patients who suffer from neuralgia, dyspepsia, indigestion and other diseases, to assist in their cure. It is, therefore, proper for them to know what *kind* of a set will prove most beneficial—a set made upon gold, or upon rubber. They can decide for themselves upon perusing this article. The writer desires to bring this subject before them through the medium of the INDEPENDENT PRACTITIONER, read as it is by physician and dentist. I shall here endeavor to place the relative merits of the two materials before them, so that they may decide from a physiological as well as a practical standpoint what is best to advise their patients. Dentists all over the country disagree on this subject, but here we shall have the facts as well as I can understand them after several years' thought on the matter. Now then, if the physician merely suggests a new set of teeth, nine cases out of ten a rubber set will be furnished, and right here let me say, it is indeed a deplorable and lamentable fact, that while the dentistry is endeavoring to raise its standard, and to elevate it to a higher plain, so as to place it side by side with all other specialties of medicine, yet in the very face of this, a great majority of dentists will advocate the use of a material for the construction of artificial dentures which is totally and absolutely unfit for such a purpose, as I will further along endeavor to show. Some of my professional brethren will doubtless say I am assuming too much; I am making too broad an assertion, as rubber has been used these many years, and has given entire satisfaction, &c.; but they shall stand each upon its own merits, and I shall not claim for one more than is its due, nor shall I condemn the other further than physiological and practical laws will allow. In the first place, how is it possible for the dental profession to expect to elevate itself as a whole, when some dentists advise, and use themselves, a material that is not only unfit, but is so simple of manipulation that an apt boy being around some dental office a few months can mount a set of teeth upon it, and as soon as they can do this, off they go with a few pairs of forceps, and at some country cross-roads or town, it may be, swing their sign in glaring letters, "Dr. John Smith, *Surgeon Dentist;*" when if they had to mount a set on gold plate, or on any other metal plate they would not attempt it. This is the lowest class, and we find them, I think, almost everywhere. But there is also a second class; it comprises those who use this material from necessity; they don't know how to make a set of teeth on gold, and if perchance they do know the principle they don't put it into practice because it is so much easier to use rubber, and it is so *cheap.* Yes, this is the only thing to my mind that it has to recommend it, *its cheapness.* This, doubtless, has a great deal to do with its general use. There is also a third class. I allude to those who use both the materials; men who were trained to the use of gold, who worked it in the days long gone by, when mechanical dentistry was honorable to the dentist, and a blessing to his patients. Now, why do this class of practitioners use it? I guess the only answer is, simply because others use it, and the patient wants it too, because *Cousin Sarah* has a set and she likes it so much; had a gold set once, but discarded it for this *nice*, *pretty rubber* set that fits so nicely, and why? Simply because she had a gold plate that did not fit; but gold was not the cause of it, it was the fault of the unskilled or careless gold *worker*; and so the old practitioner drifts along with the current, eases his conscience by the weight of his pocket-book and leaves his patients to drag out, it may be, a miserable existence. I am not alone in this position, but some of the most eminent men in the dental profession to-day are advocating the same cause. I know a professor of mechanical dentistry in one of our colleges who instructs all his students in regard to this matter particularly; he tells them without hesitation that rubber is unsuitable for artificial dentures and gives them sound reasons for it. Now then, for some of the reasons for justifying my course and opinions as well as that of many others. First, we will consider the subject from a physiological standpoint, and then from a practical view. Why is gold then better than rubber? I answer, it is a conductor of heat, cold and of electricity, while rubber is a non-conductor. Let us take this point first. We all know that the oral cavity is lined throughout with a mucous membrane filled with little follicles or minute glands that secrete and excrete a fluid, and are penetrated by a quantity of arteries, veins, lymphatics and nerves. A vulcanized rubber plate that fits the mouth accurately closes the mouths of all these little glands, and by creating great heat, irritates them and causes the entire mucous membrane which it covers to become congested and assume a hyperæmic appearance. The morbid influence, at times, is exerted to such an extent that the patient is forced to take the plate out of the mouth and go without it for several days in order to bring about a healthy condition of the parts; especially is this condition observable in the gums, they look red and inflamed, and any one who will take the trouble to examine a mouth when a plate of this kind is worn will invariably find it as represented above. The secretions are seldom if ever good, and no one can doubt that healthy secretions are necessary to perfect health. Now this condition is never brought about under a good fitting gold plate, or metal plate of any kind now in use. Why is this objectionable result not brought about under a gold plate? Simply because it is a good conductor of thermal changes, and having a perfectly smooth surface, is therefore non-irritating. So much for the conductivity and non-conductivity of the respective materials. Now, as to their composition, we all know what gold is, so it is not necessary to discuss its purity, but what is vulcanizable rubber? What substances are used in the manufacture of this material? We shall see: caoutchouc, sulphur and vermilion. Here we have a substance entering into its composition that is poisonous; vermilion, the coloring matter in vulcanite is sulphuret of mercury levigated; and its proportionate quantity mixed with the other two substances, English deep red and American Hard Rubber Company's red, contain by weight, two parts sulphide caoutchouc and one part of vermilion; sometimes white oxide of zinc or white clay is added to produce opaque, or pink rubber. Some, I am aware, argue that the presence of vermilion in this preparation can produce no injurious action in the mouth surrounded as it is by the acids and alkalies, but I will not prolong this article by discussing this question; all who read it can satisfy themselves by a little thought and reflection. I will, though, ask this question: How is it that some patients cannot even wear amalgam fillings in their teeth? Because of the mercury used in mixing up the "paste." I have seen patients whose gums were salivated just from this cause.

Now if the little mercury used in amalgam can produce such a result when it is not even in contact with the soft tissues of the mouth, what must be the effect of the sulphuret of mercury contained in a rubber plate that covers the roof, and of a lower plate that is continually bathed in the secretions? You can answer this question for yourselves. (My reference here to the mercury being extracted from the amalgam plugs and producing salivation does not, of course, apply to all individuals, by any means; but to those susceptible to mercurial salivation such a result is not uncommon.) Now let us consider the comfort which the patient experiences in wearing the two. I assert that a rubber plate cannot be kept perfectly clean, inodorous, and free from a disagreeable taste, without its being thoroughly cleansed two or three times a day; this has to be done in order to remove the mucus secretions which find lodgment upon the inner surface of the plate and adhere closely in consequence of the heat produced, and the roughness *always* left upon the inner surface. It is very seldom that a patient will take the care necessary to keep a plate of this kind perfectly clean, and when they do, it consumes much time and is, to say the least of it, some little trouble. In addition to this they occupy much more room in the mouth, as they have to be made quite thick in order to be made strong, and in inserting a new piece, it must necessarily require much more time and patience on the part of the patient, in learning to talk with them. How is it with a gold plate? If it is thoroughly cleansed upon retiring each night, it is all that is necessary. It will then at all times be inodorous and tasteless, unoffensive every way, as both the inner and the outer surfaces are perfectly smooth except, of course, for the inequalities of the mouth create little or no heat, and can be made to fit as accurately and adhere as firmly to the mouth as its monster rival, rubber. Teeth in sections can be used, the lining and soldering done so perfectly that it is just as impossible for food to get between the joints as it is in the other. Gold costs more in the beginning, I admit, but in nine cases out of ten a person who can afford to have a set of teeth, can pay for a gold plate, but as long as rubber is peddled around through the country as it is by quacks and all classes of pretenders, and made up in first-class dental offices by first-class men, so long will the general public want it. Do away with the *supply*, and there will soon be no demand. Hence, I say, if it is our desire to elevate the standard of our profession, let us endeavor to do so first by educating the people up to what it is, and in using the best material for bringing about the best results; consider first our patients, then ourselves; if they say they cannot afford a gold plate, give them a substitute in silver, at the same price as the rubber. It is better, notwithstanding its one objectionable feature, that of discoloring in the mouth. What must the public think of us as a profession, when they can take up the *New York Herald* or almost any large newspaper and see advertisements therein:—"Beautiful Sets of Teeth on rubber for only five dollars." Can you, my brother practitioners, conceive of anything more calculated to lower our standard than this? Why, it is reducing our profession to a mere trade. It has been a good thing for Josiah Bacon, but a sad thing for the dental profession. It has proved more a curse than a blessing, by great odds.

Thousands of good teeth are being sacrificed every day to this monster in sheep's clothing. Do I exaggerate in this? Not by any means, for I am fully assured that thousands of teeth that could have been saved by reputable dentists have been condemned to the forceps by the John Smiths I have before alluded to. They can make a rubber set of teeth when they could not preserve a single natural tooth. Dentistry is a healing art, as much so as medicine; it is a branch from the same stem. The same laws which govern other organs of the body preside over the teeth, and one diseased organ exerts a morbid influence over the others. The dental surgeon should understand these general laws, and unless he does he cannot expect to be called a medical man, and be recognized as such by the medical profession.

We are all laboring to attain the same result: the alleviation and cure of human suffering.

In closing, let me say that I am honest in my convictions in regard to what I have written on this subject, and if I have said anything to offend the dignity of any member of my profession I trust that a mantle of charity will be thrown over it.

